# Ameliorate Effects of Li-Fu Formula on IL-6-Mediated Cardiac Hypertrophy in Hamsters Fed with a Hyper-Cholesterol Diet

**DOI:** 10.1093/ecam/neq066

**Published:** 2011-03-10

**Authors:** Yi-Chang Cheng, Chieh-His Wu, Wei-Wen Kuo, James A. Lin, Hsueh-Fang Wang, Fuu-Jen Tsai, Chang-Hai Tsai, Chih-Yang Huang, Tsai-Ching Hsu, Bor-Show Tzang

**Affiliations:** ^1^Emergency Department of Taichung Veterans General Hospital, China Medical University, Taiwan; ^2^Department of Biological Science and Technology, China Medical University, Taiwan; ^3^Graduate Institute of Basic Medical Science, China Medical University, Taiwan; ^4^Department of Food and Nutrition, Huang-Kuang University, Taiwan; ^5^Graduate Institute of Chinese Medical Science, China Medical University, Taiwan; ^6^Department of Pediatrics, Medical Research and Medical Genetics, China Medical College Hospital, Taiwan; ^7^Department of Healthcare Administration, Asia University, Taiwan; ^8^Department of Health and Nutrition Biotechnology, Asia University, Taiwan; ^9^Institute of Immunology, School of Medicine, Chung Shan Medical University, Taichung, Taiwan No. 110, Sec. 1, Jianguo N. Road, Taichung 402, Taiwan; ^10^Clinical Laboratory, School of Medicine, Chung Shan Medical University Hospital, Taichung, Taiwan. No. 110, Sec. 1, Jianguo N. Road, Taichung 402, Taiwan; ^11^Department of Biochemistry, School of Medicine, Chung Shan Medical University, Taichung, Taiwan. No. 110, Sec. 1, Jianguo N. Road, Taichung 402, Taiwan; ^12^Institute of Biochemistry and Biotechnology, Chung Shan Medical University, Taichung, Taiwan. No. 110, Sec. 1, Jianguo N. Road, Taichung 402, Taiwan

## Abstract

Hypercholesterolemia diets are considered as major sources to cause cardiac hypertrophy. This study intends to evaluate the effects of Li-Fu formula on cardiac hypertrophy induced by hypercholesterolemia diet. Twenty-four male Golden Syrian hamsters were randomly divided into control, cholesterol and Li-Fu formula groups and fed with different experimental diets for 2 months. Histopathological analysis and western blotting were performed to measure the myocardial architecture, and various cardiac hypertrophy-associated molecules in the excised left ventricle from hamsters. The ratios of whole heart weight/body weight (BW) and left ventricle weight/BW were significantly higher in the cholesterol group but significantly lower in the Li-Fu formula group. The protein levels of both atrial natriuretic peptide and brain natriuretic peptide were significantly increased in the cholesterol group but significantly reduced in the Li-Fu formula group. Additionally, significantly increased interleukin-6, STAT3, MEK5, p-ERK5 and non-cardiomyocyte proliferate signal molecules such as p-MEK and p-ERK, were detected in the cholesterol group but significantly reduced in the Li-Fu formula group. Notably, no significant variations of inflammatory signaling molecules, including p-P38 and p-JNK, were detected in all groups. Our experimental results demonstrated the significant reductions of cardiac hypertrophy and related eccentric hypertrophy signaling, non-cardiomyocyte proliferate signaling in the excised left ventricle of hamsters from the Li-Fu formula. We suggested the protective effects of Li-Fu formula on cardiac hypertrophy that may be useful in prevention or treatment of hypertrophy-associated cardiovascular diseases.

## 1. Introduction

Hypercholesterol diets are the major cause of cardiac hypertrophy [1]. Cardiac hypertrophy is recognized as a cardiac adaptive response to any stress that can exist in a state of compensation or progress to a decompensated state over time [2]. Prolonged hypertrophy of the cardiomyocytes is demonstrated as the main cause of sudden cardiac death [3]. A number of studies indicated that various diseases have been associated with cardiac hypertrophy including occlusive atherosclerotic coronary heart disease (CHD), associated myocardial infarction (MI), heart failure hypertension, endocrine disorders, toxicants and bacterial endocarditis [4–7].

Atrial natriuretic peptide (ANP) and brain natriuretic peptide (BNP) are known as the cardiac hormones in normal adults that were secreted by the atria and ventricles. Higher levels of ANP and BNP expression are expressed in the fetal ventricles than adult ventricles [8]. Cardiac ANP and BNP levels are increased in MI of animal models [9], heart failure [10], hypertrophy [11] and also in human cardiac diseases [12]. Increased expressions of ANP and BNP are observed in ventricular during the molecular process of cardiac hypertrophy, which are recognized as markers of the induction of the embryonic gene program in ventricular hypertrophy [13].

Interleukin (IL)-6 is known as a potent hypertrophic factor of cardiomyocytes [14, 15]. The IL-6 receptor system consists of various signaling pathways including inflammatory-related p38 MAPK, hypertrophy involved STAT1-STAT3 heterodimer pathway, STAT3 homodimer pathway and non-cardiomyocyte proliferative related MAPK extracellular signal-regulated kinase (ERK)s pathway that are activated by the dimerization of gp130 [16–19]. The activation of STAT3-dependent signaling pathway by gp130 was reported to promote cardiac myocyte hypertrophy [20], herein the STAT1 and the STAT3 were shown to be chronically phosphorylated in the failing heart [21]. Moreover, the ERK5 molecule plays a critical role in post-natal eccentric hypertrophy of the heart [9, 22]. ERK5 and its upstream MAPK-kinase 5 (MEK5) reveals a specific role in transduction of cytokine signals that regulate serial sarcomere assembly and in the induction of eccentric cardiac hypertrophy resulting in dilated cardiomyopathy and sudden death [22]. Therefore, it is crucial to investigate the pathologic role of IL-6-MEK5-ERK5 signaling pathway under cardiac hypertrophy. Additionally, various molecules have been elucidated responsible for the development of cardiac hypertrophy, including mitogen activated protein kinase (MAPK), phosphoinositide 3-kinase (PI3K) and calcineurin pathway [23]. The extracellular-regulated kinase (ERK), the c-Jun N-terminal kinases (JNK) and the p38 MAPK cascades (p38) enrolled in the MAPK pathway also play crucial roles in the development of cardiac hypertrophy [24].

To avoid the side effects by administration of western drugs, growing studies were performed to investigate the natural products for cardiac protection that have been used as drugs or diet supplements for a long history in many medical experiences. Recent studies reported the cardioprotective effect of various oriental herb extracts or dietary supplements including *Fructus crataegi, Salvia miltiorrhiza* and *Astragali radix*. The quercetin is the main ingredient in *Fructus crataegi* that has been demonstrated as an anti-inflammatory substance by inhibiting TNF-*α* release from macrophages and recognized to have cardiac protective effect [25, 26]. *Salvia miltiorrhiza* is mainly composed of sodium tanshinone IIA sulfonate (STS), a derivative of tanshinone IIA that can reduce myocardial infarct size and prolong the survival cardiac cell in rabbit and human [15, 27, 28]. *Astragali radix* contains many isoflavones, isoflavonoids and many saponins, which have been demonstrated to have protective effects on heart by reducing inflammation, oxidant and cardiac ischemia-reperfusion injury [29–34] In our recent publication, we also demonstrated the protective effect of Li-Fu formula composing of celery, black fungus, mushroom, *Saliva miltior rhiza, Crataegi cuneata* and *Astragali radix* on cardiac apoptosis [35].

To further understand the effects and possible mechanisms of Li-Fu formula on cardiac hypertrophy, we examined the expression of hyper-trophic associated molecules in the cardiac tissues from hamsters that were fed with hypercholesterol diets and suggested the cardiac protective effects of Li-Fu formula by reducing the cardiac hypertrophy.

## 2. Methods

### 2.1. Animals and Diet

Male Golden Syrian hamsters weighting 135–170 g at the age of 8 weeks were purchased from National Laboratory Animal Center, Taipei, Taiwan, and housed in an animal room at 22 ± 2°C with a 12/12 h light-dark cycle under supervision of Institutional Animal Care and Use Committee of China Medical University, Taichung, Taiwan. Hamsters were acclimatized for 2 weeks while receiving free access to water and were fed chow diet (Lab Diet 5001; PMI Nutrition International Inc., Brentwood, MO, USA) *ad libitum*. For each experiment, 24 hamsters were randomized into three groups as control, cholesterol and Li-Fu formula groups and switched to experimental diets. Three independent experiments were performed. The control, cholesterol and Li-Fu formula groups received chow diet, chow diet with 0.2% cholesterol (Sigma, St Louis, MO, USA), and chow diet with 0.2% cholesterol and 2% Li-Fu formula for 8 weeks, respectively. Celery and Black fungus were obtained from common supermarket and mushroom, *Saliva miltior rhiza, Crataegi cuneata* and *Stragali radix* were purchased from traditional Chinese pharmacy. To make Li-Fu formula, every component of desired weight was crushed and mixed with a blender, then placed in 1000 ml distilled water and boiled for 1 h under reflux. The resultant solution was divided into several parts and stored in a –80°C freezer for further use [35]. The Li-Fu formula is composed of celery, black fungus, mushroom, *Saliva miltior rhiza, Crataegi cuneata* and *Astragali radix* as shown in Table 1 and the experimental dietary composition is shown in Table 2. Diets were prepared weekly and stored at −80°C. All experimental procedures were performed according to the NIH Guide for the Care and Use of Laboratory Animals. All protocols were approved by the Institutional Animal Care and Use Committee of China Medical University, Taichung, Taiwan. Food intake and food spillage were measured daily, and body weight was recorded every 3 days. 

### 2.2. Cardiac Characteristics

Three groups of hamsters at age of 8-9 months were weighed and decapitated after receiving 8 weeks of experimental diets. The hearts of animals were excised and cleaned with distilled H_2_O. The left and right atrium and ventricle were separated and weighed. The BW, left ventricle weight (LVW), the ratios of the whole heart weight (WHW) to BW and the ratios of the LVW to BW, were measured and calculated.

### 2.3. Hematoxylin-Eosin Staining

The hearts of animals were excised and were soaked in formalin and covered with wax. Slides were prepared by deparaffinization and dehydration. They were passed through a series of graded alcohols (100, 95 and 75%), 15 min each. The slides were then dyed with hematoxylin. After gently rinsing with water, each slide was then soaked with 85% alcohol, 100% alcohol I and II for 15 min each. At the end, they were soaked with Xylene I and Xylene II. Photomicrographs were obtained using Zeiss Axiophot microscopes.

### 2.4. Tissue Extraction

Cardiac tissue extracts were obtained by homogenizing the left ventricle samples in a PBS buffer (0.14 M NaCl, 3 mM KCl, 1.4 mM KH_2_PO_4_, 14 mM K_2_HPO_4_) at a ratio of 100 mg tissue/0.5 ml PBS for 5 min. The homogenates were placed on ice for 10 min and then centrifuged at 12 000 g for 30 min. The supernatant was collected and stored at –70°C for further experiments. Protein concentration was determined using a BioRad Protein Assay (BioRad Laboratories, Hercules, CA, USA) and were quantified by absorbance at 595 nm using a spectrophotometer (Beckman Coulter, Palo Alto, CA, USA).

### 2.5. Western Blot

Western blotting was performed as described in our previous report [35]. Protein samples were separated in 12.5% of SDS-PAGE and electrophoretically transferred to nitrocellulose membrane (Amersham Biosciences, Piscataway, NJ). After blocking with 5% non-fat dry milk in PBS for 30 min at room temperature, antibodies against ANP, BNP, IL-6, STAT3, MEK5, p-EKR5, MEK, p-MEK, phosphorylated ERK (p-ERK), p-P38, JNK, p-JNK and *α*-tubulin (Santa Cruz Biotechnology, Santa Cruz, CA, USA) were diluted to 1:500 with 5% nonfat dry milk in PBS and then incubated for 1.5 h at room temperature. The membranes were washed twice with PBS-Tween for 1 h and incubated with second antibody conjugated with horseradish peroxidase (Promega Corp., Madison, WI, USA) for 1 h that was diluted 1000-fold with 5% nonfat dry milk in PBS. Pierce's Supersignal West Dura HRP Detection Kit (Pierce Biotechnology Inc., Rockford, IL) was used to detect antigen-antibody complexes. The blots were scanned and quantified by densitometry (Appraise, Beckman-Coulter, Brea, CA, USA).

### 2.6. Statistical Analysis

All of the statistical analyses were performed using SPSS 10.0 software (SPSS Inc., Chicago, IL). Three independent experiments were repeated. Statistical analyses were performed using the analysis of variance plus posterior multiple comparison test to test the difference. The data between two experimental animal groups was compared by Student's *t*-test for two independent samples. In all cases, a difference at *P* < .05 was considered statistically significant.

## 3. Results

### 3.1. Experimental Diets and Cardiac Characteristics

To investigate the effect of Li-Fu formula on hypertrophy in cardiac cells, we examined the BW and cardiac characteristics. First, Li-Fu formula was prepared as described in materials and methods and the compositions of the Li-Fu formula was shown in Table 1. Table 2 presents the ingredients of experimental diets for different groups of hamsters. BW, LVW, the ratios of WHW to BW and the ratios of LVW to BW of hamsters from control, cholesterol and Li-Fu formula groups were detected (Table 3). The ratios of WHW/BW and LVW/BW were significantly higher in hamsters of cholesterol group compared to control group. Notably, the ratios WHW/BW and LVW/BW were significantly reduced in the hamsters from Li-Fu formula group compared with cholesterol group (Table 3). 

### 3.2. Cardiac Architecture Changes

**Figure 1:**
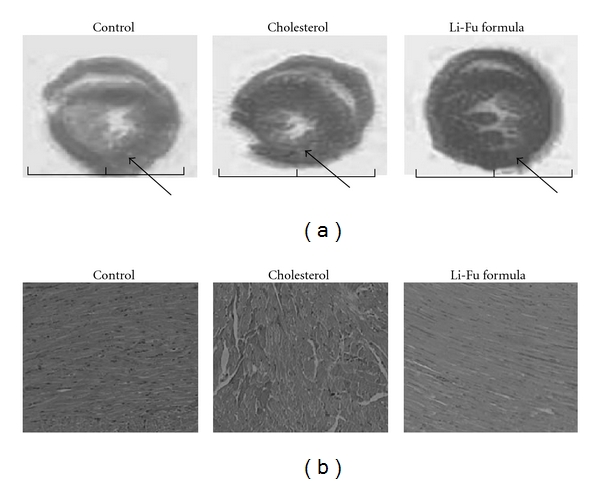
Cardiac cross sections and cardiomyopathic changes in hamsters of control, cholesterol and Li-Fu formula groups. (a) The cross section of whole heart in the three groups. Arrows indicate that the left ventricular lumen diameters increased in the cholesterol group but decreased in the Li-Fu formula group. (b) Representative histopathological analysis of cardiac tissue sections with hematoxylin and eosin staining in hamsters of control, cholesterol and Li-Fu formula groups. The images of myocardial architecture were magnified by 100 times.

**Figure 2:**
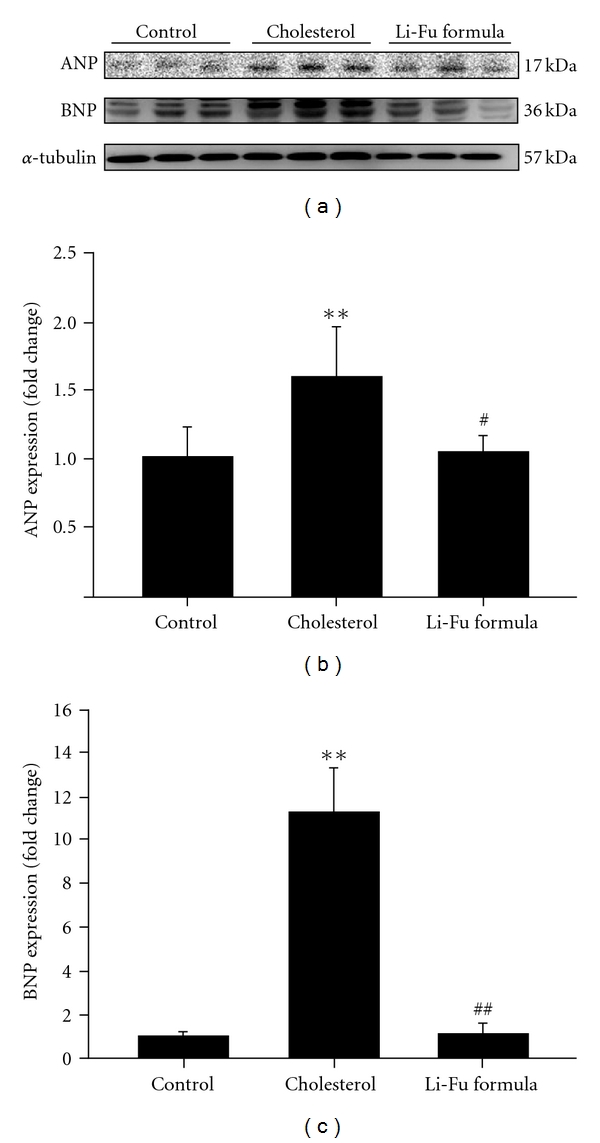
(a) The representative protein products of ANP and BNP extracted from the left ventricles of excised hearts in hamsters of control, cholesterol and Li-Fu formula groups were measured by western blotting analysis. ((b), (c)) Bars represent the relative protein quantification of ANP and BNP on the basis of *α*-tubulin. All bars indicate mean values ± SD (*n* = 6 in each group). **P < .01, significant differences between control and cholesterol group. ^#^ *P* < .05 and ^##^ *P* < .01, significant differences between cholesterol and Li-Fu formula groups.

To further confirm the effect of Li-Fu formula on cardiac hypertrophy, we performed cross-sectional analysis of whole heart and histopathological analysis of ventricular tissue stained with hematoxylin and eosin. We found that ventricular wall thickness significantly increased in the cholesterol group, but decreased significantly in the Li-Fu formula group (Figure 1(a)). The ventricular myocardium in control group showed normal architecture with normal interstitial space. In contrast, the irregular myocardial architecture and the increased interstitial space were observed in cholesterol group that showed structural disorganization and cardiomyocyte disarray. However, these abnormal architectures were significantly decreased in Li-Fu formula group compared with cholesterol group (Figure 1(b)). Moreover, the protein levels of both ANP and BNP were significantly increased in hearts of cholesterol group compared to control group. In contrast, significantly reduced ANP and BNP protein expressions were detected in hearts of hamsters from Li-Fu formula group (Figure 2). 

### 3.3. Effect of Li-Fu Formula on Cardiac Hypertrophy-Associated Signaling Pathways

**Figure 3:**
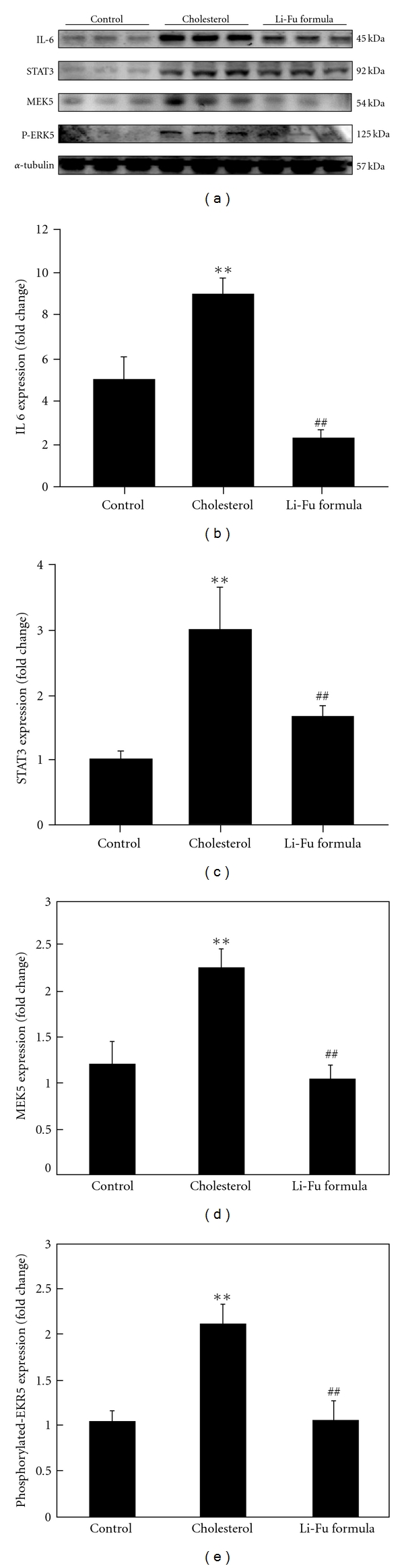
(a) The representative protein products of IL-6, STAT3, MEK5 and p-ERK5 extracted from the left ventricles of excised hearts in hamsters of control, cholesterol and Li-Fu formula groups were measured by western blotting analysis. ((b)–(e)) Bars represent the relative protein quantification of IL-6, STAT3, MEK5 and p-ERK5 on the basis of *α*-tubulin. All bars indicate mean values ± SD (*n* = 6 in each group). **P < .01, significant differences between control and cholesterol groups. ^##^ *P* < .01, significant differences between cholesterol and Li-Fu formula groups.

**Figure 4:**
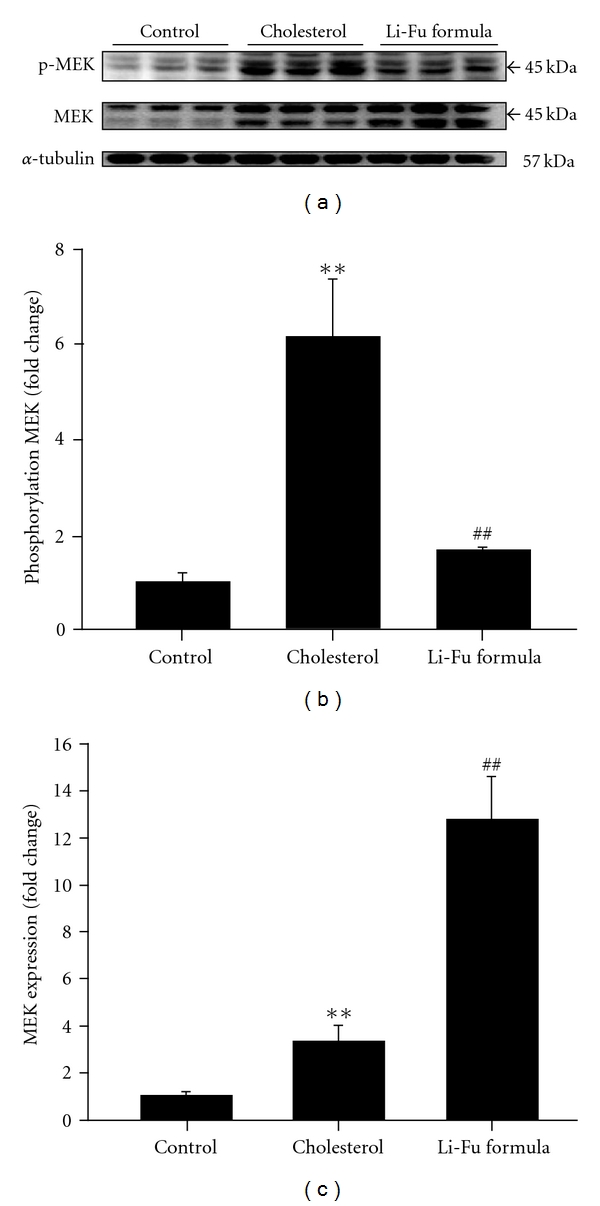
(a) The representative protein products of p-MEK and MEK extracted from the left ventricles of excised hearts in hamsters of control, cholesterol and Li-Fu formula groups were measured by western blotting analysis. ((b), (c)) Bars represent the relative protein quantification of p-MEK and MEK on the basis of *α*-tubulin. All bars indicate mean values ± SD (*n* = 6 in each group). **P < .01, significant differences between control and cholesterol groups. ^##^ *P* < .01, significant differences between cholesterol and Li-Fu formula groups.

**Figure 5:**
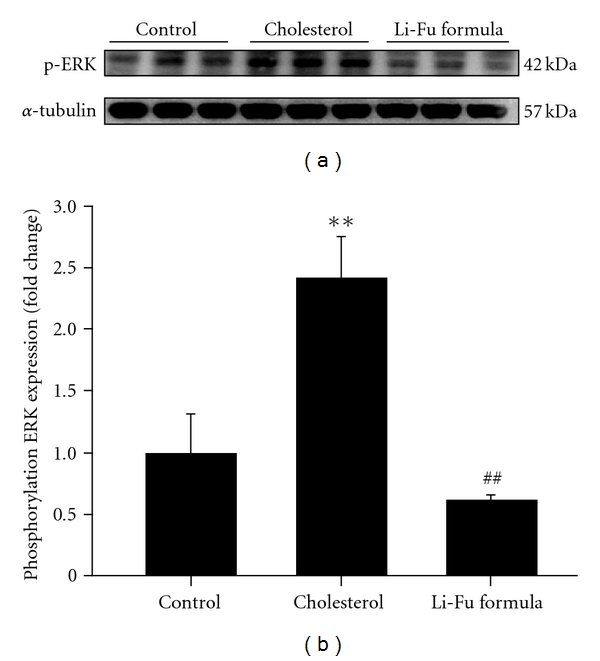
(a) The representative protein product of p-EKR extracted from the left ventricles of excised hearts in hamsters of control, cholesterol and Li-Fu formula groups were measured by western blotting analysis. (b) Bars represent the relative protein quantification of p-ERK on the basis of *α*-tubulin. All bars indicate mean values ± SD (*n* = 6 in each group). **P < .01, significant differences between control and cholesterol groups. ^##^ *P* < .01, significant differences between cholesterol and Li-Fu formula groups.

In order to identify the hyper-trophic factor IL-6, signal transducer and activator of transcription STAT-3 and mitogen-activated protein kinase/ERK (MEK) signaling pathways that were associated with the cardiac hypertrophy induced by hypercholesterol diet, the protein products of IL-6, STAT3, MEK5 and p-ERK5 were measured by western blotting. In hearts from the cholesterol group, the protein products of IL-6, STAT3, MEK5 and p-ERK5 were significantly increased compared to the hearts of control group (Figure 3). However, significantly decreased IL-6, STAT3, MEK5 and p-ERK5 protein expression was observed in hearts of Li-Fu formula group (Figure 3). We further detected the protein levels of MEK and p-MEK. As shown in Figure 4, significantly increased MEK and p-MEK protein levels were detected in hearts of cholesterol group compared to control group (Figure 4). Notably, significantly decreased p-MEK was observed in hearts of Li-Fu formula group compared to cholesterol group (Figure 4). Additionally, significantly increased p-ERK protein was detected in hearts of cholesterol group compared to control group. In contrast, significantly reduced p-ERK protein was observed in hearts of hamsters from Li-Fu formula group compared to those from cholesterol group (Figure 5). However, no significant variations in p-P38 and p-JNK protein levels were detected among all experimental groups (data not shown). 

## 4. Discussion

Hypercholesterolemia diets have been recognized as the major cause of cardiac hypertrophy and are associated with various heart diseases [4, 5, 7, 32]. Our experimental results indicate the significant reduction of the WHW/BW and LVW/BW ratios in hamsters from Li-Fu formula group compared with those from cholesterol group. Moreover, the hypertrophic marker protein such as ANP, BNP, eccentric hypertrophic-related factors such as IL-6, STAT3, MEK5, p-ERK5, p-MEK and p-ERK were significantly increased in cholesterol group, whereas significant reduction of all these proteins were observed in Li-Fu formula group.

**Figure 6:**
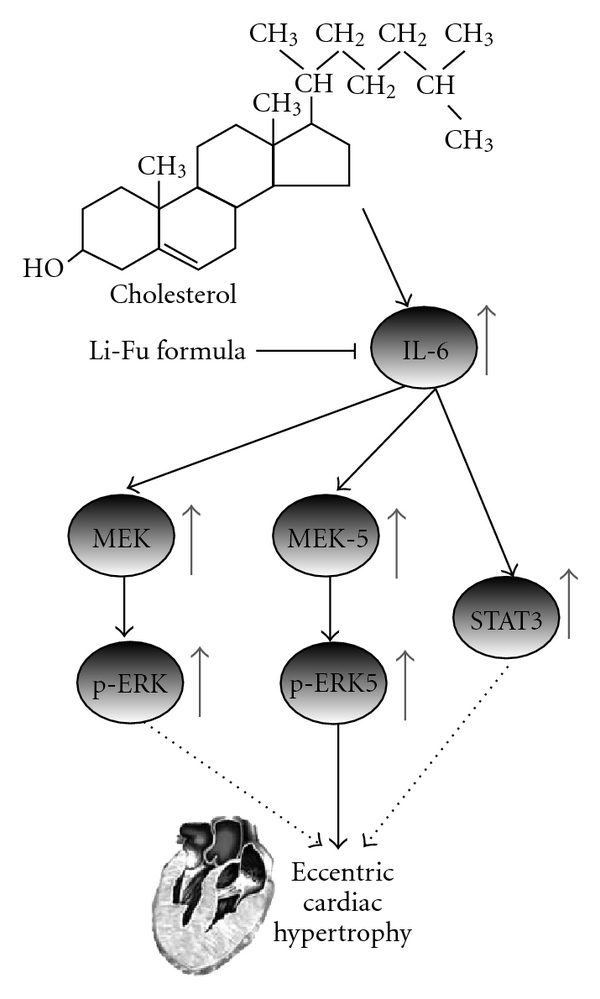
Our proposed hypothesis that cardiac IL-6, MEK-5-ERK-5 and STAT3 hypertrophic pathways and MEK1/2-ERK1/2 non-cardiacmyocyte proliferative pathway are more activated in hyper cholesterol-fed hamster hearts. The eccentric hypertrophy-related pathway, IL-6-related MEK5-ERK5 pathways and MEK1/2-ERK1/2 non-cardiacmyocyte proliferative pathway may play a part of role for developing eccentric cardiac hypertrophy and pathological changes in hyper cholesterol-fed hamster hearts. Dash lines represent possible theoretical pathways but are still unconfirmed. Up arrows and down arrows on the right side represent increases and decreases, respectively.

The interleukin (IL)-6 is known as a pleiotypic factor that has been associated with various cardiac diseases [14, 36, 37]. Elevated IL-6 mRNA is observed in patients of cardiac hypertrophy with hypertrophic cardiomyopathy [37]. As shown in Figure 6, STAT3 and MAPK (ERK)s pathway were induced by IL-6 receptor signaling systems and contributed to the cardiac hypertrophy [1, 9]. Accordingly, our experimental results revealed significant elevation of IL-6 expression in the excised ventricle of hamsters from Cholesterol group as well as those hypertrophic related signaling molecules including STAT3, MEK5, p-ERK5. Notably, the significant reduction of these hypertrophic factors and signaling molecules were detected in the excised ventricle of hamsters from Li-Fu formula group. To further clarify the involved signaling pathway, we further examined the MAPK pathway that consists three major cascades including the non-cardiomyocyte proliferative ERK, and the inflammatory related c-Jun N-terminal kinases (JNK), and the p38 MAPK cascades (p38) [24]. As revealed in current study, significant increase of p-ERK was observed in the excised ventricle of hamster from Cholesterol group and the p-ERK level was significantly reduced in the excised ventricle of hamster from Li-Fu formula group. Moreover, higher increase of p-MEK, the upstream kinase activator of EKR, was also detected in hamsters from Li-Fu group compared to cholesterol group. However, no significant differences in protein levels of p-P38 and p-JNK were detected between hamsters from cholesterol and Li-Fu formula groups. Although the precise mechanisms of Li-Fu formula on reducing IL-6 expression merit further investigations, these findings indeed demonstrated that Li-Fu formula has the effect against cardiac hypertrophy by attenuating non-cardiomyocyte proliferation related p-ERK cascade but not P38 or JNK cascade. 

Because of the moderated side effects than Western drugs, more than half of the population in the world relies on traditional medicine for therapeutic needs. Indeed, herbal remedies and alternative medicines are used throughout the world and in the past herbs often represented the original sources of most drugs [38–40]. The Li-Fu formula was first created by Dr Li-Fu Chen, China Medical University, Taichung, Taiwan, and composed of various dietary supplements and oriental herbs, including celery, black fungus, mushroom, *Saliva miltior rhiza, Crataegi cuneata* and *Astragali radix* that were routinely used as traditional medicine in oriental worlds. For instance, a major ingredient of Li-Fu formula, *Salvia miltiorrhiza*, is known as “Danshen" and mainly composed of sodium tanshinone IIA sulfonate (STS), a derivative of tanshinone IIA that is also known to protect cardiovascular ischemia-reperfusion and oxidant injuries [15, 27, 28, 30, 32–34, 39, 40]. To elucidate the effect and possible mechanism of Li-Fu formula on hypercholesterolemia-induced cardiac hypertrophy, we performed the histopathological analysis and western blotting to measure the myocardial architecture, and expression of different cardiac hypertrophy-associated molecules in the excised left ventricle from hamsters. Notably, markedly reduced ratios of WHW/BW and LVW/BW were observed in hamsters from Li-Fu formula group compared with those from cholesterol group. These findings did suggest the protective effects of Li-Fu formula on cardiac hypertrophy.

In the world, more than half of the population relies on traditional medicine for therapeutic needs either by stewing or solution extracting [39–41]. Although the precise mechanism of most herbal medicine or dietary supplement has not been fully understood, the experience of the traditional use over the years cannot be neglected. Altogether, our experimental results revealed that Li-Fu formula, the traditional oriental herbs and diet supplements formula have significant protective effects against cardiac hypertrophy. Besides the attenuated expression of ANP and BNP, the effect against cardiac hypertrophy of Li-Fu formula is probably via the reduction of eccentric hypertrophy related IL-6 receptor pathway and non-cardiomyocyte proliferation involved ERK signaling cascade but not JNK and P38 cascades.

## Funding

Grant CMU96-100 from the China Medical University, Taichung, Taiwan.

## Figures and Tables

**Table 1:** Composition of Li-Fu formula.

Items	Weight (g)	Portion (%)
Celery^(a)^	25	2.5
Black fungus^(b)^	320	32.3
Mushroom^(c)^	420	42.4
Saliva miltior rhiza	75	7.6
Crataegi cuneata	75	7.6
Stragali radix	75	7.6

^(a)^Celery is also kanown as *Apium graveolens.*

^(b)^Black fungus indicates Wood ear, or pinyin: mù ěr, lit. “wood ear" or “tree ear" are commonly sold in Asian markets as dietary supplement.

^(c)^The standard for the name “mushroom" is the cultivated white button mushroom, *Agaricus bisporus*.

**Table 2:** Formulation and calculated composition of diets.

Ingredients (% wt/wt)	Control diet	Cholesterol diet	Experimental diet
Chow diet,	99.5	99.3	97.3
(Rodent 5001)			
Soybean oil			
	0.5	0.5	0.5
Cholesterol	0	0.2	0.2
Li-Fu formula	0	0	2

**Table 3:** WHW, LVW, WHW to BW, and LVW to BW ratio.

	Control	Cholesterol	Li-Fu formula
Whole heart weight (mg)	410.6 ± 36.10	423.1 ± 21.92	377.1 ± 2.40
LVW (mg)	290.5 ± 8.82	292.0 ± 12.98	269.1 ± 10.10
WHW/BW^(a)^	3.8 ± 0.03	3.9 ± 0.01*	3.6 ± 0.03^*#*^
LVW/BW^(a)^	2.7 ± 0.04	2.8 ± 0.01*	2.6 ± 0.03^*#*^

^(a)^Data present in percentage.

*P < .05, versus control.

^*#*^ *P* < .05, versus cholesterol.
